# Supplementary material for: The Primary Cilium and its Hedgehog Signaling in Nociceptors Contribute to Inflammatory and Neuropathic Pain
Source: Res Sq. 2024 Feb 26:rs.3.rs-3812442. Preprint. [Version 1] doi: 10.21203/rs.3.rs-3812442/v1 (PMC10925437; doi:10.21203/rs.3.rs-3812442/v1)
Supplement: 1 [file NIHPPRS3812442V1-supplement-1.pdf]

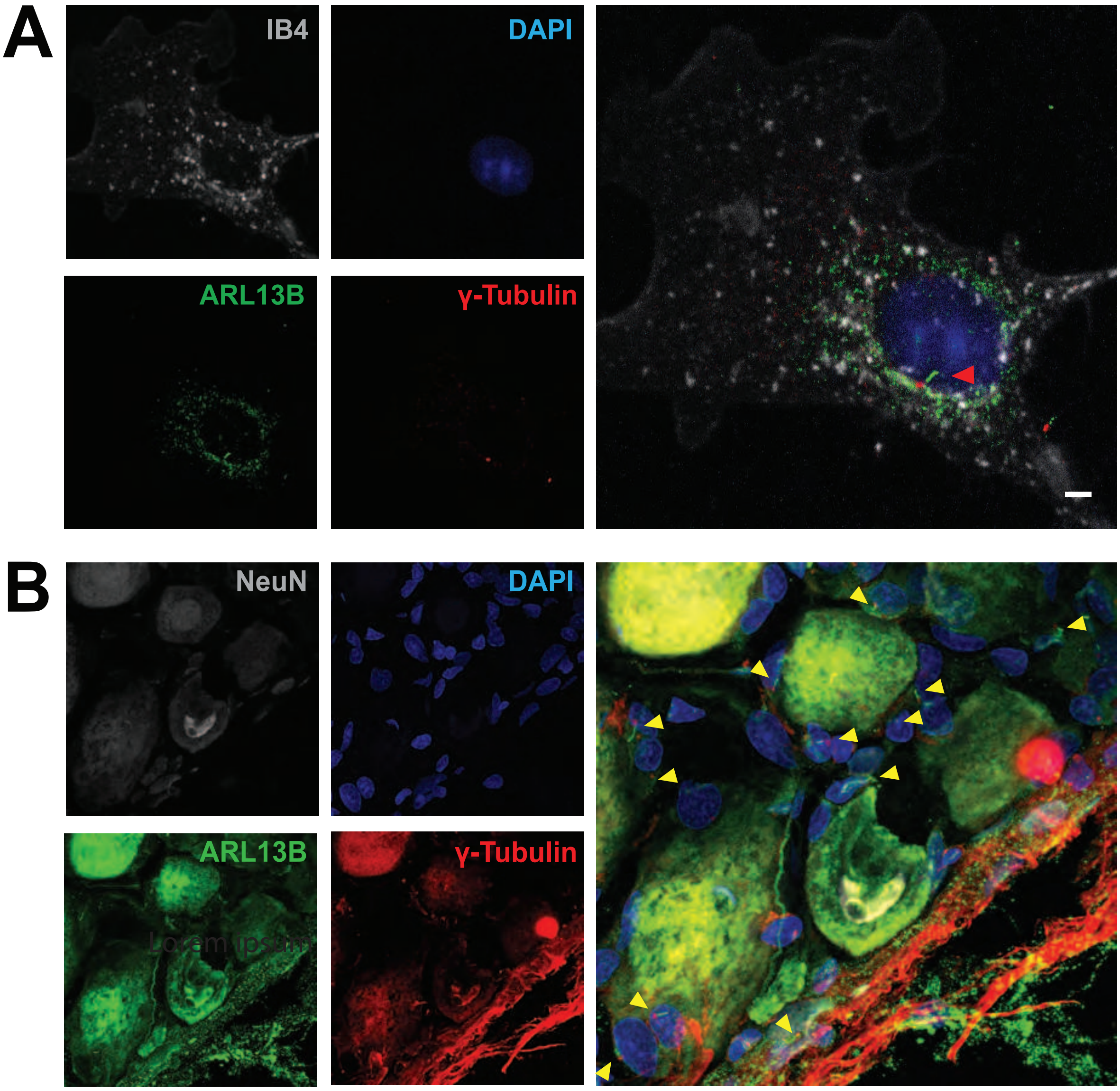

Supplementary Figure 1. Mouse DRG neurons elaborate primary cilia in vitro, and satellite cells are also ciliated. (A, B) Immunohistofluorescence analysis of acutely dissociated adult mouse DRG culture (A) and of adult rat DRGs *in vivo* (B). Coverslips (A) and histological sections (B) were labeled with antibodies recognizing ARL13B (green), gamma-tubulin (red), and Fox-3 (B, NeuN, greyscale). (A) IB4 staining is indicated in greyscale. (A, B) Cell nuclei marked by DAPI (blue). Red and yellow arrowheads indicate neuronal and non-neuronal primary cilia, respectively. Scale bar: 3 μm.
